# Strengthening one health systems: A readiness assessment across Croatia, Ghana and Nigeria

**DOI:** 10.1016/j.onehlt.2026.101533

**Published:** 2026-07-28

**Authors:** Fadi El-Jardali, Racha Fadlallah, Francis Sena Nuvey, Nadine Darwiche, Barbara Bekavac, Pavle Jeličić, Luka Delak, Brigita Hengel, John Humphrey Amuasi, Leslie Mawuli Aglanu, Daniel Opoku, Jesse Uneke, Onyedikachi Chukwu, Amos Enyi, Davi Romão, Tanja Kuchenmüller, Anna Fahrion

**Affiliations:** a Health Management and Policy, American University of Beirut, Beirut, Lebanon; b Knowledge to Policy Center, American University of Beirut, Beirut, Lebanon; c Friedrich-Loeffler-Institut, Südufer 10, 17493 Greifswald-Insel Riems, Germany; d Croatian Institute of Public Health, Rockefellerova 7, Zagreb, Croatia; e Croatian Agency for Agriculture and Food, Kardinala Alojzija Stepinca 17, Osijek, Croatia; f Kumasi Centre for Collaborative Research in Tropical Medicine, Kwame Nkrumah University of Science and Technology, Kumasi, Ghana; g Bernhard Nocht Institute of Tropical Medicine, Hamburg, Germany; h University Medical Centre Hamburg-Eppendorf, Hamburg, Germany; i University Medical Centre Groningen, University of Groningen, Groningen, the Netherlands; j Technische Universität Berlin, Berlin, Germany; k African Institute for Health Policy & Health Systems II, David Umahi Federal University of Health Sciences, Uburu, Ebonyi State University, Abakaliki, Nigeria; l Veredas Institute, São Paulo, Brazil; m Social Research Institute, University College London, 20 Bedford Way, London, United Kingdom

**Keywords:** One health assessment, One health governance, Multisectoral collaboration, Evidence-informed policymaking, Health system readiness, System strengthening, Croatia, Ghana, Nigeria, Assessment tool

## Abstract

**Background:**

Despite widespread endorsement, many countries continue to face challenges operationalizing One Health. Existing assessment tools are often fragmented and limited in scope, focusing on specific technical functions (e.g., surveillance, coordination, or prioritization) or diseases (e.g. AMR, zoonotic disease) and rarely integrate governance, environmental health, evidence-informed policymaking and strategic prioritization within a single systems assessment. The Knowledge- to-Policy (K2P) One Health System Readiness Assessment (KOHSRA) tool addresses this gap by integrating a comprehensive systems-level readiness assessment across the human-animal-environment interface with structured priority setting, enabling countries to translate assessment findings into strategic priorities for policy and implementation.

**Methods:**

Within the One Health-Evidence-Informed Policy Approaches (OH-EVI) project, we piloted KOHSRA in Croatia, Ghana and Nigeria. The tool employed a standardized, facilitated national self-assessment process, comprising two phases: Assessment and Prioritization. The assessment phase combined documentary review, stakeholder mapping and multisectoral consensus workshops across five components: (1) One Health system foundation; (2) One Health risk profile; (3) Policy coherence and One Health integration; (4) Evidence-informed policymaking for One Health; and (5) One Health stakeholder landscape. The prioritization phase synthesized assessment findings into country-specific SWOT profiles, which informed the identification of strategic priorities for policy and implementation.

**Results:**

All three countries demonstrated foundational One Health capacities including sectoral institutions, technical expertise, surveillance arrangements and coordination mechanisms, but these remained insufficiently institutionalized within routine national systems and varied in maturity. Common cross-country gaps included fragmented governance and multisectoral coordination, the absence of dedicated domestic financing, weak surveillance interoperability, limited environmental integration, and insufficient mechanisms for evidence synthesis and knowledge translation. The prioritization phase translated these findings into context-specific strategic priorities, with recurring themes including strengthened institutional governance and multisectoral coordination, integrated surveillance across the human-animal-environment interface, sustainable financing, workforce development and One Health education, AMR control, and climate resilience.

**Conclusions:**

KOHSRA represents a practical framework that can support countries in assessing national One Health system readiness and identifying context-specific priorities for policy and implementation. Its application in Croatia, Ghana, and Nigeria identified common system readiness gaps and context-specific priorities for strengthening national One Health systems. Future applications in additional countries and formal validation studies are warranted to strengthen the evidence base supporting its broader applicability.

## 1 Introduction

The world is facing increasingly interconnected health challenges, including emerging zoonotic diseases, antimicrobial resistance (AMR), environmental degradation, food safety threats and climate change. These challenges and other emerging disease threats increasingly transcend the boundaries between human, animal, and environmental health, requiring integrated approaches to governance and policymaking [1].

The One Health approach recognizes the interdependence of human, animal, and environmental health and has emerged as an important framework for addressing these complex challenges [2]. The One Health High-Level Expert Panel (OHHLEP) defines One Health as “an integrated, unifying approach” that aims to sustainably balance and optimize the health of people, animals, and ecosystems [3]. In addition to strengthening health security, One Health contributes to broader Sustainable Development Goals (SDGs), particularly those related to health, food security, water and sanitation, climate action and ecosystem sustainability [4], [5].

Despite growing international commitment to One Health, many countries continue to face substantial barriers to its operationalization. These include fragmented governance, weak multisectoral coordination, inadequate data-sharing systems, insufficient financing and limited policy coherence across sectors [6], [7], [8]. Although international frameworks provide broad guidance for One Health policy and implementation, structured tools that comprehensively assess national One Health system readiness remain limited [9], [10].

Existing tools generally focus on specific diseases, technical functions or operational domains rather than overall system readiness. For example, the One Health Zoonotic Disease Prioritization (OHZDP) [11] and the One Health Systems Assessment for Priority Zoonoses (OHSAPZ) tools [12] focus on the prioritization and assessment of zoonotic diseases. Other tools address specific functions, including multisectoral coordination through the OH-SMART [13], surveillance and information sharing through the Surveillance and Information Sharing Operational Tool (SIS-OT) [14], and alignment between human and animal health systems through the National Bridging Workshops (NBWs) [15]. Many also do not adequately address all three components of the One Health triad (human, animal and environmental health), with environmental health frequently absent or underrepresented despite the growing influence of climate change, biodiversity loss, ecosystem degradation, and pollution on health risks and the emergence of zoonotic diseases [16], [17].

Beyond technical capacity, effective One Health implementation also depends on institutions that can generate, synthesize and apply evidence to policymaking [18]. However, One Health research translation frameworks remain limited, and existing assessment tools primarily emphasize system assessment rather than translating findings into policy priorities and implementation actions [18], [19].

Consequently, countries often rely on multiple disconnected instruments to assess different dimensions of One Health readiness. A recent global review similarly concluded that existing assessment tools remain insufficient for addressing governance, financing, environmental determinants, and long-term system strengthening [20].

Recognizing the need for a more integrated assessment of national One Health system readiness, the Knowledge to Policy (K2P) Center at the American University of Beirut developed the K2P One Health System Readiness Assessment (KOHSRA) Tool by building on and extending existing One Health tools and frameworks. KOHSRA combines a systems-level readiness assessment with structured priority setting within a single participatory process to support country-led One Health systems strengthening. Unlike many existing One Health assessment tools, KOHSRA integrates multiple dimensions of system readiness including governance, institutional capacity, financing, evidence-informed policymaking, and stakeholder engagement. Additionally, it explicitly recognizes the human-animal-environment interface by integrating environmental health across multiple assessment domains, including One Health risk profiling, policy coherence, multisectoral governance, and stakeholder representation, thereby supporting a more balanced assessment of national One Health systems. Importantly, the tool extends beyond assessment by incorporating a structured prioritization phase that translates assessment findings into strategic priorities for policy and implementation.

The application described in this paper was conducted within the broader One Health-Evidence-Informed Policy Approaches (OH-EVI) project, implemented by the Friedrich-Loeffler-Institut (FLI) in collaboration with the World Health Organization's Evidence-Informed Policy Network (WHO EVIPNet). The OH-EVI project aimed to strengthen evidence-informed policymaking (EIP) and knowledge translation in Croatia, Ghana and Nigeria through the development of Evidence Briefs for Policy and structured policy dialogues on priority One Health issues. As a foundational component of the project, the KOHSRA Tool was applied to assess national One Health system readiness and identify context-specific priorities to inform subsequent evidence synthesis and policy engagement processes.

This study presents the application of the KOHSRA Tool in Croatia, Ghana and Nigeria. It had a dual objective: first, to assess national One Health system readiness and identify context-specific strategic priorities; and second, to examine the operational feasibility and potential utility of KOHSRA as a structured framework for multisectoral One Health assessment across diverse implementation settings.

## 2 Methods

### 2.1 Development and structure of the KOHSRA Tool

The KOHSRA Tool was developed through an iterative multi-stage process comprising: (i) a review of international One Health frameworks, (ii) benchmarking against existing operational assessment tools, (iii) technical expert review, and (iv) review by country leads from the three participating countriest.

Key One Health frameworks and guidance documents, including the World Bank Operational Framework [10], the European Centre for Disease Prevention and Control One Health Framework for Action [21], the Washington State Department of Health One Health Needs Assessment Report [20] and the inventory of One Health tools [22] were reviewed to identify the core domains required for operationalizing national One Health systems. These informed the conceptual foundation of the KOHSRA tool (Supplementary File 1). The emerging assessment framework was then benchmarked against existing assessment tools (OHZDP, OH-SMART, NBW, and SIS-OT) to identify complementarities, minimize duplication and determine where broader systems-level coverage was needed (Supplementary File 1). This process informed the final assessment components, domains and indicators. To further address the limitations of existing assessment tools, a structured prioritization phase (Phase II) was incorporated to translate assessment findings into strategic priorities for policy and implementation.

The draft tool underwent technical review by five One Health and evidence-to-policy specialists from the Friedrich Loeffler Institute (FLI) and WHO EVIPNet, who were selected based on their complementary expertise in One Health, evidence-informed policymaking, and health systems as well as their roles within the OH-EVI project. The review focused on the comprehensiveness, relevance and clarity of the proposed domains and indicators. Because the tool was designed as an applied, systems-level assessment framework to support policy and implementation, rather than as a psychometric measurement instrument, formal psychometric validation procedures were not undertaken. Expert feedback informed revisions to domain definitions and indicator wording. Key refinements included clearer separation of policy and legislative frameworks from governance arrangements and institutional capacity; greater emphasis on financing and sustainability as a distinct subdomain; more explicit integration of the human-animal-environment interface within Component 1; and stronger attention to evidence-informed policymaking.

Country leads (*n* = 3) from Croatia, Ghana and Nigeria subsequently reviewed the framework for contextual relevance and feasibility. Their feedback informed minor refinements to indicator wording and implementation guidance, while the core framework remained unchanged. Specific revisions included simplifying the stakeholder assessment through the use of existing stakeholder structures and sectoral representation instead of formal power analysis, and replacing composite scoring with domain-level interpretation.

The final KOHSRA tool consists of two interlinked phases: Assessment and Prioritization (Table 1). The Assessment phase comprises five components, each encompassing multiple domains: (1) One Health System Foundations; (2) One Health Risk Profile; (3) Policy Coherence and One Health Integration; (4) Evidence-Informed Policymaking (EIP) for One Health; and (5) One Health Stakeholder Landscape. The Prioritization phase uses assessment findings and country-specific SWOT profiles to identify strategic priorities for policy and implementation. The tool generates both diagnostic findings and actionable priorities, linking multisectoral assessment to strategic decision-making. Although primarily designed for national-level application, it may be adapted to subnational contexts to examine how national policies and institutional arrangements are operationalized at regional or district levels. The full tool is available from the authors upon request.

**Table 1:** Overview of KOHSRA Tool.

Phases	Component	Key Domains	Assessment and Analysis Approach
Phase I: Assessment	Component 1. One Health System Foundations	Policy and legislation; Governance and institutional capacity; Financing and sustainability; Workforce development and training; Surveillance and response; Research and knowledge systems; Community engagement; Monitoring and evaluation (M&E) and learnings.	Structured indicator-based assessment: Each domain comprises predefined indicators assessed using standardized categorical response scales (e.g., yes/no; high/moderate/low/none; effective/partial/ineffective). Ratings are determined through facilitated stakeholder consensus and supported by narrative justification and documentary evidence where available.
	Component 2. One Health Risk Profile	Public health risks; Animal health risks; Environmental risks; Socio-economic determinants.	Descriptive risk profiling: National and international data sources are synthesized alongside stakeholder input to characterize priority One Health risks, trends, and contextual vulnerabilities. No formal indicator scoring is applied
	Component 3. Policy Coherence and One Health Integration	One Health policies; Public health policies; Environmental policies; Agricultural policies.	Qualitative policy analysis: Relevant policies and legislation are reviewed to assess cross-sectoral coherence, legal mandates, implementation gaps, and alignment with One Health principles. Findings are synthesized narratively across sectors.
	Component 4. Evidence-Informed Policymaking (EIP) for One Health	Evidence synthesis; Deliberative processes; Knowledge brokering and translation.	Qualitative institutional assessment: Existing evidence-informed policymaking capacities are examined through structured qualitative questions exploring evidence synthesis, deliberative processes, knowledge brokering, and translation mechanisms. Findings are synthesized narratively.
	Component 5. One Health Stakeholder Landscape	Stakeholder mapping; Institutional alignment.	Stakeholder mapping and institutional analysis: Stakeholders are characterized according to sectoral representation, institutional roles and engagement with existing One Health structures to identify participation gaps and opportunities for multisectoral collaboration.
Phase II: Strategic Prioritization		SWOT analysis; Identification of strategic priorities for policy and implementation.	Assessment findings from Components 1-5 are synthesized into country-specific SWOT profiles, which inform the identification of potential strategic actions and priorities. Stakeholders then review and prioritize these through facilitated deliberation guided by predefined prioritization criteria. No numerical scoring or formal ranking is undertaken.

### 2.2 Implementation approach

The KOHSRA Tool is designed as a facilitated national self-assessment that emphasizes stakeholder engagement and contextualized analysis to promote shared ownership and strengthen institutional commitment to One Health. The assessment is conducted by a multidisciplinary working group comprising representatives from the human, animal and environmental health sectors, coordinated by a national focal lead. Additional experts may be included as needed to ensure comprehensive sectoral representation. Prior to the assessment, a desk review of relevant national policies, legislation, strategies, reports and other supporting documents is undertaken to inform discussions and provide evidence for the assessment.

The tool was piloted in Croatia, Ghana and Nigeria between September 2024 and February 2025 as part of the broader OH-EVI project. In each country, the assessment was integrated into existing OH-EVI multisectoral working groups established to support country-specific Evidence Briefs for Policy. Implementation comprised a desk review of national legal and policy documents; stakeholder mapping and confirmation of multisectoral representation; multisectoral workshop(s) to complete the readiness assessment and priority-setting exercise; and synthesis and validation of findings to produce a consolidated national readiness profile and strategic priorities. The same version of the KOHSRA tool was used across all three countries to support consistency of implementation and cross-country comparison.

A total of 26 stakeholders participated in Croatia, 26 in Ghana, and 60 in Nigeria across the assessment and validation processes. The relatively larger number in Nigeria reflected a broader national-level validation dialogue involving additional stakeholders. Participants included representatives from relevant ministries, public health institutes, veterinary services, environmental agencies, academia and other key One Health stakeholders, depending on the country context. For each assessment domain, participants reviewed the available evidence and discussed their perspectives before reaching consensus through facilitated deliberation. Where available, documentary evidence was used to support the assessments. When differing views emerged, the facilitator guided discussion to clarify interpretations and review supporting evidence in order to reach a shared assessment. No formal voting or majority-decision rules were used. To minimize institutional and social desirability bias, workshops followed standardized facilitation guidance and indicator definitions, while the multidisciplinary composition helped ensure that the assessments reflected multiple institutional perspectives rather than those of a single sector.

Following completion of the readiness assessment, findings were synthesized into a country-specific SWOT profile, from which potential strategic actions and knowledge translation priorities were identified. Participants then reviewed these potential priorities and reached consensus on the final strategic priorities. Depending on the country context and participant availability, the prioritization exercise was conducted either during the same workshop or in a subsequent session. Discussions were guided by predefined criteria, including the magnitude of identified readiness gaps, importance for strengthening national One Health systems, alignment with national goals and policy agendas, stakeholder demand, implementation feasibility, and the country-specific policy context. These criteria were intended to guide structured discussion rather than generate numerical rankings. Consensus was reached through facilitated deliberation without formal voting, numerical scoring or ranking of priorities. Potential knowledge translation priorities, including topics for country-specific Evidence Briefs for Policy, were also identified (Table 6).

The resulting national profiles, SWOT findings, and strategic priorities were subsequently reviewed and validated through country consultations. These validated country submissions formed the basis for the project-level cross-country synthesis and comparative analysis.

### 2.3 Data analysis

Each country team submitted a validated national assessment profile comprising indicator-level responses, narrative justifications, supporting documentary evidence, country-specific SWOT findings, and strategic priorities developed through the facilitated workshops and validation consultations. Indicators in Component 1 (One Health System Foundations) were assessed using predefined categorical response scales appropriate to each indicator, such as yes/no, high/moderate/low/none, and effective/partial/ineffective. The remaining components were assessed qualitatively and descriptively through documentary review and facilitated stakeholder discussion.

For cross-country comparison and visualization, responses from Components 1 and 4 were recoded into three standardized reporting categories: Available, To some extent, and Absent. For categorical indicators, the recoding reflected the meaning of the original response scale. For qualitative indicators, categories were assigned based on participants' narrative accounts and supporting evidence. These categories facilitated broad cross-country comparison while recognizing that the nature and maturity of similarly categorized arrangements could vary across countries. To preserve contextual nuance, comparisons were interpreted alongside the original responses, narrative justifications and documentary evidence.

The project team compared the validated country submissions to identify common patterns, areas of divergence, and recurring system strengths and gaps. The synthesized findings and visualizations were reviewed by the respective country teams to confirm their accuracy and contextual interpretation. No composite readiness score or statistical ranking of countries was undertaken. The findings reflect the documentary evidence and consensus judgments of participating stakeholders and were not independently verified.

## 3 Results

The findings are presented according to the two phases of the KOHSRA tool: Phase I (Assessment) and Phase II (Strategic Prioritization) (Table 1). Within Phase I, results are organized by the assessment components and domains. The narrative focuses on the principal cross-country patterns and contextually important differences, while the accompanying tables present the country-level findings and consensus classifications (where applicable).

## 4 Phase I: Assessment

### 4.1 Component 1: One Health System Foundations

Component 1 assessed the governance, institutional and operational foundations required for effective One Health implementation across the human, animal and environmental sectors. It covered policy and legislation; governance and institutional capacity; financing and sustainability; workforce development and training; surveillance and response; research and knowledge management; community engagement and public awareness; and monitoring, evaluation and learning. Across the three countries, existing sectoral institutions, technical expertise, surveillance arrangements, and emergency response systems provided an important foundation for One Health implementation. However, these capacities remained largely organized within sector-specific systems. Participants reported persistent gaps in formal institutionalization, sustainable financing, routine multisectoral coordination, integrated surveillance system across the human-animal-environment interface, interdisciplinary workforce development and environmental sector engagement (Table 2).

**Table 2:** Overview of One Health system foundations across Croatia, Ghana, and Nigeria.

Domains	Nigeria	Croatia	Ghana
**Policy & Legislation**			
National One Health policy	Absent	Absent	Absent
National One Health strategy	Available	Absent	To some extent
Integration of One Health principles into national policies	To some extent	To some extent	To some extent
Legal provisions supporting One Health initiatives (human, animal and environmental sectors)	Available	Available	To some extent
Enforcement of laws and legislations related to zoonotic diseases, AMR and environmental health	To some extent	To some extent	To some extent
**Governance & Institutional Capacity**			
Formal mechanisms for intersectoral collaboration	To some extent	Available	Available
National One Health coordinating body	Available	Absent	Available
Multi-sectoral representation in coordinating body (human, animal and environmental sectors)	Available	To some extent	Available
Leadership assigned for One Health implementation	Available	Absent	Available
**Financing and Sustainability**			
Dedicated national budget for One Health	Absent	Absent	Absent
External funding supporting One Health	Available	Available	Available
**Workforce Development and Training**			
Availability of trained multidisciplinary workforce	Available	Available	Available
Interdisciplinary One Health training opportunities	Available	To some extent	To some extent
Applied epidemiology training program	Available	To some extent	To some extent
Integration of One Health into educational curricula	To some extent	To some extent	To some extent
**Surveillance & Response**			
Integrated surveillance across human, animal and environmental sectors	To some extent	Available	To some extent
Multisectoral data sharing protocols	Absent	To some extent	To some extent
National One Health coordination platform	To some extent	To some extent	To some extent
Multi-hazard preparedness and response plan	To some extent	To some extent	To some extent
Joint outbreak response mechanisms	To some extent	To some extent	Available
Reported effectiveness of coordinated responses during health emergencies	Absent	To some extent	To some extent
**Research and Data Systems**			
National One Health research agenda	To some extent	Absent	Absent
Joint multidisciplinary research initiatives	Available	Available	Available
One health research publications	To some extent	To some extent	Absent
One Health databases and platforms	To some extent	Absent	Absent
Accessibility of One Health data across sectors	To some extent	To some extent	To some extent
**Community Engagement & Public Awareness**			
National One Health awareness programs	Available	Absent	To some extent
Community participation in One Health activities	To some extent	Absent	To some extent
**Monitoring, Evaluation & Learning**			
Integrated national One Health monitoring, evaluation and learning framework established	Absent	Absent	Absent
Disease-specific One Health monitoring targets established	Available	Available	Available
Clearly defined reporting responsibilities for One Health indicators	Absent	To some extent	Absent
Regular assessment and reporting of One Health indicators	To some extent	To some extent	To some extent
Impact evaluation of One Health initiatives	Absent	To some extent	Absent
Lessons learned from M&E documented and used for continuous One Health system improvement	To some extent	To some extent	Absent

Note: *Available* = fully present or established; *To some extent* = partially present, implemented, or functional; *Absent* = not present or no supporting evidence identified.

#### 4.1.1 Policy and legislation

One Health principles had been incorporated into sectoral policies in all three countries to varying degrees, although none had a fully adopted and enforceable national One Health policy. The level of institutionalization differed across countries.

Nigeria had previously established a National One Health Strategic Plan (2019–2023), which provided a formal basis for multisectoral collaboration. However, integration into broader national policies and implementation at subnational levels remained incomplete. Legal provisions addressing zoonotic diseases, AMR and environmental health were in place, but enforcement was considered partial.

Ghana had developed a draft One Health strategy that had not yet been formally adopted at the time of the assessment. One Health principles were partially reflected in policies relating to animal health and zoonotic diseases. However, participants identified limited legal provisions supporting integrated One Health implementation and weak enforcement of relevant zoonotic and environmental legislation.

Croatia did not have a dedicated national One Health policy or strategy at the time of assessment. Nonetheless, One Health-related principles were incorporated in EU-aligned legislation on food safety, veterinary public health, AMR and zoonotic disease control. Participants noted that these provisions operated primarily through separate sectoral frameworks rather than a unified national One Health approach.

Across all three countries, legal provisions supporting zoonotic disease control and AMR were present to varying degrees; however, implementation remained largely sector-specific, with limited legal mechanisms supporting integrated governance across the human, animal and environmental sectors.

#### 4.1.2 Governance and institutional capacity

All three country assessments identified governance structures or working arrangements supporting multisectoral collaboration, although their level of formalization and institutionalization varied. Nigeria and Ghana had formally designated national coordination mechanisms whereas Croatia relied on collaboration among existing sectoral institutions.

Nigeria's National One Health Coordinating Unit led by the Nigeria Centre for Disease Control (NCDC), provided formal leadership and an operational platform for multisectoral collaboration. However, participants reported incomplete integration across human, animal and environmental sectors and limited operationalization at subnational levels.

Ghana's One Health Technical Working Group coordinated by the National Disaster Management Organization (NADMO), brought together representatives from the human, animal and environmental sectors. However, participants identified weak legal institutionalization and limited engagement of finance and economic-planning institutions.

Croatia relied on existing sectoral structures among the Ministries of Health, Agriculture, and Environment together with public health and veterinary institutions, without a dedicated One Health coordinating body or formally assigned leadership. Coordination was considered functional for specific issues but fragmented in the absence of an overarching national governance mechanism.

All three countries faced challenges in achieving sustained and integrated coordination across sectors, particularly in strengthening routine collaboration beyond emergency response and ensuring greater participation of environmental agencies within national governance structures.

#### 4.1.3 Financing and sustainability

None of the three country assessments identified a dedicated national budget line for One Health. Nigeria and Ghana relied primarily on development partners and donor-supported programs whereas Croatia benefited from EU funding opportunities. Although these funding mechanisms enabled implementation of several One Health activities, participants considered the absence of predictable domestic financing a major constraint to sustainable implementation, national ownership and long-term institutionalization. The limited participation of finance and economic-planning institutions further constrained integration of One Health priorities into routine government planning and budgeting.

#### 4.1.4 Workforce development and training

All three countries demonstrated technical capacity relevant to One Health across the public health, veterinary, and environmental sectors.

Croatia showed comparatively stronger institutional capacity in veterinary public health, food safety, zoonotic disease surveillance, AMR and laboratory systems, reflecting long-established regulatory and technical structures aligned with EU requirements. Ghana and Nigeria had established Field Epidemiology Training Programmes (FELTPs) including the Nigeria Field Epidemiology and Laboratory Training Programme (NFELTP) and the Ghana FELTP, alongside broader initiatives such as the Regional Disease Surveillance Systems Enhancement (REDISSE) Program that strengthened applied epidemiology and outbreak response capacity.

Operational veterinary service capacity also differed across countries. Croatia maintained comparatively well-developed veterinary infrastructure supported by routine vaccination programs, established disease-control systems and effective collaboration between public and private veterinary services. In contrast, Ghana and Nigeria reported workforce shortages, infrastructure constraints, and logistical limitations, particularly in rural areas, reducing capacity for routine animal health service delivery, vaccination, and veterinary disease surveillance.

Despite these existing capacities, interdisciplinary One Health education remained only partially institutionalized within higher education, with most training remaining discipline-specific and primarily concentrated within public health and, to a lesser extent, veterinary sciences. Environmental professionals participated in One Health activities but were less consistently integrated into interdisciplinary education and workforce development initiatives.

#### 4.1.5 Surveillance and response

Surveillance and emergency response systems were established in all three countries, providing an important foundation for outbreak detection and response. However, their level of integration across the human, animal and environmental sectors varied.

Croatia was characterized as having comparatively stronger surveillance arrangements, supported by 10.13039/501100000780EU reporting requirements, standardized surveillance systems, established veterinary and food safety surveillance and sectoral data-sharing mechanisms. However, participants reported that coordination was generally stronger during outbreaks than as a routine One Health function, and environmental information remained only partially integrated into surveillance activities.

Both Ghana and Nigeria maintained functional but more fragmented surveillance arrangements. Ghana had joint outbreak-response teams and a national multi-hazard preparedness framework; however, surveillance systems remained fragmented with limited routine cross-sectoral data-sharing protocols and largely reactive coordination. In addition, the National Action Plan for Health Security had expired at the time of the assessment and was identified as requiring revision. Nigeria had partially integrated surveillance for selected zoonotic diseases together with joint outbreak-response arrangements across ministries. Participants identified weaknesses in routine information sharing, limited interoperability of surveillance systems, incomplete implementation at the state level and insufficient integration of environmental information into preparedness and response activities.

Across the three countries, surveillance systems functioned more effectively during outbreaks than as routine integrated One Health platforms. Environmental surveillance and ecosystem monitoring were identified as the least integrated components of national surveillance systems, limiting comprehensive assessment of environmentally driven health threats.

#### 4.1.6 Research and knowledge systems

Across the three countries, research and data systems remained fragmented, characterized by the absence of a comprehensive national One Health research agenda and centralized platforms for coordinating and sharing knowledge across the human, animal and environmental sectors.

In Croatia, research was mainly supported through EU projects and collaborations with the WHO and the Food and Agriculture Organization (FAO), but these activities remained largely issue-specific, and participants did not identify a centralized One Health knowledge management platform at the time of assessment.

Ghana's research institutions conducted studies on COVID-19, Monkeypox, AMR and related topics, but research remained fragmented with no centralized platform for coordinating or sharing findings. Participants also reported limitations in the availability and reliability of national datasets aligned with One Health indicators.

In Nigeria, research collaborations addressed Lassa fever and other priority diseases. And while data platforms such as DHIS2 and the National Animal Disease Information System (NADIS) were available, participants noted fragmented datasets, inconsistent accessibility and limited integration across human, animal and environmental sectors.

Overall, the findings indicate that research and information systems remained largely sector-specific, limiting the generation and management of integrated One Health knowledge across sectors.

#### 4.1.7 Community engagement and public awareness

The assessment suggested comparatively stronger nationally coordinated public awareness and community outreach initiatives in Nigeria. Participants in Nigeria highlighted national One Health awareness campaigns, One Health Day activities, cross-ministerial engagement, collaboration with non-governmental organizations and the media, and the involvement of risk communication teams. However, a more comprehensive national communication strategy was considered necessary to improve the consistency and impact of outreach efforts. Ghana relied primarily on institution-led awareness activities, including World Rabies Day, World Antimicrobial Resistance Awareness Week, International One Health Day events, and informal engagement through the One Health Platform WhatsApp group. However, these efforts lacked national coordination. Croatia had not implemented national One Health public awareness campaigns, and outreach remained largely confined to technical and academic circles, with communication beyond professional sectors limited to disease-specific messaging during outbreaks.

Across all three countries, community engagement activities were identified but remained largely disease-specific, occurring primarily during disease outbreaks or awareness campaigns. Public engagement on broader One Health concepts including prevention, environmental stewardship, and the interconnectedness of human, animal, and ecosystem health remained limited.

#### 4.1.8 Monitoring, evaluation and learning

monitoring, evaluation and learning relevant to One Health remained largely embedded within disease-specific or sectoral programs rather than integrated national One Health frameworks.

Croatia followed EU-established M&E frameworks for zoonotic disease control, with clearer reporting roles and regular disease-specific monitoring. However, One Health indicators were usually embedded within broader public health and veterinary reports rather than assessed independently, and dedicated national One Health performance reviews remained limited.

In Nigeria, monitoring and evaluation activities were embedded within sectoral plans, with disease-specific targets for conditions such as tuberculosis, brucellosis, and Ebola. However, reporting roles were unclear, assessments remained irregular, and monitoring was largely confined to public health and, to a lesser extent, animal health indicators, limiting accountability and system-wide coordination. Furthermore, impact evaluations were reported as rare, with lessons learned documented mainly through occasional After-Action Reviews and NCDC platforms.

In Ghana no national One Health monitoring and evaluation framework was identified through the assessment. Monitoring remained largely project-specific, particularly for AMR and fragmented across institutions with limited attention to environmental indicators. Assessments were sporadic, often led by external partners, with no structured evaluation of One Health impact or formal system for capturing and applying lessons learned.

Despite these established sectoral monitoring and evaluation systems, integrated national One Health monitoring and evaluation remained underdeveloped across all three countries. Environmental indicators and cross-sectoral One Health outcome measures were seldom incorporated, limiting the assessment of integrated impacts across the human–animal–environment interface and reducing opportunities for institutional learning.

### 4.2 Component 2: One Health Risk Profiles

This component examined national risk profiles across four dimensions: public health risks, animal health risks, environmental risks, and socio-economic determinants (Table 3). Although the three countries differed in their epidemiological and socioeconomic contexts, several common patterns emerged. Across all three countries, One Health risks extended beyond infectious disease threats to encompass environmental change, livestock production systems, climate-sensitive hazards and socioeconomic conditions that influence opportunities for disease emergence and transmission. These findings highlight the importance of addressing interacting human, animal, and environmental drivers when setting national One Health priorities.

#### 4.2.1 Public health risks

The three countries exhibited distinct public health risk profiles. Croatia faced relatively low burdens of priority zoonotic diseases but increasing concern regarding antimicrobial resistance (AMR) and climate-sensitive diseases. Ghana experienced recurrent zoonotic outbreaks, including rabies and anthrax, together with a high AMR burden (∼88% multidrug-resistant Gram-negative infections). Nigeria faced recurrent outbreaks of priority zoonoses, including Lassa fever and Mpox, alongside widespread antimicrobial misuse and limited information on several zoonotic diseases.

#### 4.2.2 Animal health risks

Animal health risks reflected differences in livestock production systems and human–animal interactions. Croatia's regulated livestock sector reduced many risks, although transboundary animal diseases and wildlife interactions remained important. Ghana's rapid agricultural expansion and predominantly low-biosecurity livestock production increased opportunities for zoonotic transmission. Nigeria faced persistent endemic animal diseases, agricultural expansion, and extensive human–animal contact, particularly in rural communities.

#### 4.2.3 Environmental risks

Environmental drivers were recognized as important contributors to One Health risks across all three countries. Croatia's risk profile was influenced by climate-sensitive diseases and localized land-use change. Ghana faced increasing pressures from agricultural expansion and ecosystem degradation, while Nigeria experienced widespread deforestation, biodiversity loss, pollution, and environmental degradation that heightened the potential for zoonotic spillover.

#### 4.2.4 Socioeconomic determinants

Socioeconomic conditions further shaped national risk profiles. Geographic disparities persisted in Croatia despite universal healthcare coverage. In Ghana and Nigeria, rural livelihoods, subsistence farming, occupational animal contact, unequal access to health services, and limited health literacy increased vulnerability to One Health threats.

**Table 3:** Country-level One Health risk profiles.

Domain	Croatia	Ghana	Nigeria
Public Health Risks	Endemic and emerging infectious disease risks remain under surveillance, with AMR an increasing public health concern (national AMR index: 36.6%). Climate-sensitive and vector-borne diseases continue to pose localized public health risks.	Recurrent zoonotic outbreaks (e.g., rabies and anthrax), fragmented epidemiological data and a high AMR burden (**∼**88% MDR in Gram-negative infections) represent major public health threats particularly in underserved areas.	Recurrent outbreaks of priority zoonoses (e.g., Lassa fever and mpox), widespread antimicrobial misuse, and limited data on other zoonotic diseases contribute to a complex public health risk profile.
Animal Health Risks	Livestock production is supported by established veterinary systems, although transboundary animal diseases and wildlife-related risks require continued vigilance.	Rapid agricultural expansion, predominantly low-biosecurity livestock production, and frequent human–animal interactions increase opportunities for zoonotic disease transmission.	Extensive livestock production, persistent animal diseases (e.g., rabies and bovine tuberculosis), and close human–animal interactions increase the potential for zoonotic spillover, particularly in rural communities. Vaccination rates are low,
Environmental Risks	Strong environmental regulation mitigates many risks, although climate-sensitive diseases, land-use changes and localized ecosystem disturbances remain important drivers of One Health threats.	Rapid agricultural expansion, low-biosecurity livestock practices and environmental degradation create zoonotic hotspots and increase exposure to zoonotic and other One Health threats.	Widespread deforestation, pollution, and biodiversity loss raise zoonotic spillover risks. Weak integration of environmental surveillance with health systems limits early detection of emerging threats.
Socio-economic Determinants	Universal healthcare provides broad service coverage, although geographic disparities persist in remote and underserved areas. Socio-economic vulnerabilities continue to affect exposure and response to health threats.	Rural areas face major access, education, and information gaps. Subsistence farming and high-risk jobs increase exposure to zoonotic threats. Inequities weaken health system responsiveness.	Poverty, limited healthcare access, low health literacy and poor education reduce disease reporting. Subsistence farming increases close human–animal contact and subsequent exposure to zoonotic diseases and reduced response capacity.

### 4.3 Component 3: Policy Coherence and One Health Adoption

Component 3 assessed the coherence of sectoral policies with One Health principles and the extent to which policies across the human health, animal health and environmental sectors supported a coordinated One Health approach. While all three countries had relevant sectoral policies, policy coherence remained limited across sectors.

Croatia did not have a dedicated national One Health policy; however One Health principles were reflected across existing sectoral policies. Public health policies addressed zoonotic disease surveillance, AMR control, vector management, and public awareness while environmental and agricultural policies emphasized climate adaptation, biodiversity protection, sustainable livestock practices, and biosecurity. Nevertheless, these policies operated largely within separate sectoral frameworks, limiting coherence across the broader One Health agenda.

Ghana had made comparatively greater progress toward policy coherence through the development of a draft national One Health strategy covering surveillance, food safety, environmental health, and transboundary risks. The 2020 National Health Policy prioritized epidemic preparedness and disease surveillance, while environmental and agricultural policies emphasized conservation, climate change, food security, and livestock disease management. Participants, nonetheless, noted implementation gaps and incomplete alignment under a common One Health framework.

Nigeria exhibited the least coherent policy environment. No dedicated One Health policy had been adopted at the time of assessment. Existing public health policies did not adequately address zoonotic diseases, AMR, or food safety within a coherent national framework. Environmental and agricultural policies remained largely siloed, with limited attention to animal health and weak linkages across human, animal and environmental health sectors.

Across the three countries, relevant human-health, animal-health, agricultural and environmental policies existed, but coherence across these policy domains was limited. Environmental and climate-related policies were generally developed and implemented separately from health and veterinary policies, hindering the translation of sectoral commitments into coordinated One Health implementation.

### 4.4 Component 4: Evidence-Informed Policymaking for One Health

This component focused on institutional capacity for evidence-informed policymaking by examining how evidence was synthesized, interpreted, deliberated, and translated to support One Health policy development and multisectoral decision-making. It comprised three domains: evidence synthesis deliberative engagement and knowledge brokering and translation (Table 4).

#### 4.4.1 Evidence synthesis

Across the three countries, participants reported that evidence relevant to One Health was generated by multiple institutions but remained fragmented across sectors. None of the countries had a centralized repository or institutional mechanism for synthesizing and integrating evidence across the human, animal and environmental sectors. Nigeria and Ghana faced additional challenges related to the limited availability of integrated One Health information. Across all three settings, environmental evidence was least represented in evidence synthesis efforts.

#### 4.4.2 Deliberative engagement

Participants in Nigeria and Ghana reported limited institutionalization of regular, structured platforms for regular engagement and deliberation among policymakers and stakeholders from human-, animal- and environmental-health sectors. This constrained sustained joint agenda setting, interpretation of evidence and collaborative policy design. Croatia had established platforms for cross-sectoral deliberation, largely aligned with EU standards, providing stronger institutional support for stakeholder engagement and collaborative agenda setting.

#### 4.4.3 Knowledge brokering and translation

Formal mechanisms for translating One Health evidence into policy-relevant products were characterized as limited across all three countries. Nigeria did not have a dedicated One Health knowledge brokering institution identified through the assessment although some policy analysis functions were undertaken within government and partner-supported programs. Ghana similarly lacked institutions specifically mandated to translate One Health research into policy. Croatia had some policy analysis activity within government departments and academia, but participants did not identify a formalized One Health knowledge brokering structure encompassing human, animal and environmental sectors.

Across all three countries, knowledge translation remained largely project-driven and dependent on individual institutions rather than institutionalized national mechanisms.

**Table 4:** Country-level assessment of capacity for evidence-informed policymaking for One Health.

Domains	Nigeria	Ghana	Croatia
**Evidence Synthesis**			
Centralized One Health evidence repository	Absent	Absent	Absent
Institutional mechanisms for One Health evidence synthesis and integration	To some extent	To some extent	To some extent
**Deliberative Engagement**			
Institutionalized mechanisms or platforms for multisectoral deliberative engagement of policymakers and stakeholders	Absent	Absent	To some extent
**Knowledge Brokering & Translation**			
Institutional arrangements for knowledge brokering and translation to support One Health policymaking	Absent	Absent	Absent
Institutional capacity for One Health policy analysis	To some extent	Absent	To some extent

Note: *Available* = fully established or consistently implemented; *To some extent* = partially established, inconsistently implemented, or present with important gaps; *Absent* = not identified or no supporting evidence available.

### 4.5 Component 5: One Health Stakeholder Landscape

This component assessed the stakeholder landscape and institutional relationships supporting One Health implementation across Croatia, Ghana and Nigeria. All three countries had broad stakeholder networks spanning the human, animal, environmental, agricultural, academic and development sectors. However, participants reported that stakeholder engagement and collaboration remained uneven, with environmental institutions, finance and planning authorities, local governments, civil society organizations and communities less consistently engaged in governance and priority-setting processes.

Croatia had a relatively well defined institutional landscape with clear sectoral responsibilities across the Ministries of Health, Agriculture, and Environmental Protection and Green Transition together with public health, veterinary, and food safety institutions. However, participants noted the absence of an overarching national mechanism to coordinate these stakeholders under a unified One Health approach.

Ghana's stakeholder landscape included government ministries, technical agencies, academia, and international organizations, supported by a draft One Health strategy and an active technical working group. Despite broad stakeholder representation, institutional cohesion and sustained multisectoral collaboration remained limited.

Nigeria had a complex stakeholder landscape involving federal and state institutions, technical agencies, academia, and development partners. Although formal One Health coordination structures were in place, participants identified overlapping mandates, persistent sectoral silos, limited engagement of environmental actors and continued reliance on externally supported initiatives.

Across the three countries, broad stakeholder representation did not consistently translate into effective multisectoral collaboration. Common challenges included fragmented institutional relationships, uneven stakeholder participation, and limited engagement beyond the health and agriculture sectors.

## 5 Phase II: Prioritization

### 5.1 SWOT Analysis

Findings from Components 1–5 were synthesized into country-specific SWOT profiles to support the identification of potential priority areas for strengthening One Health systems.

The SWOT analysis revealed differences in institutional capacity and system readiness across Croatia, Ghana, and Nigeria. While all three countries had established existing multisectoral coordination structures, common weaknesses included fragmented routine coordination, unsustainable financing, insufficient surveillance integration and limited sharing of data across sectors. The nature and severity of these challenges, however, differed by country. Common opportunities included international collaboration and regional learning, workforce development, research partnerships, access to external funding and technical networks, and the use of emerging technologies. Key threats varied across countries and included political transitions or instability, climate-related health risks, antimicrobial resistance, insecurity, and infrastructure and rural service-delivery constraints (Table 5).

**Table 5:** Country-level SWOT profiles.

	Ghana	Croatia	Nigeria
Strengths	Draft national One Health strategy; multisectoral coordination mechanism; experience in outbreak response; strong research capacity; availability of policy briefs & strategic documents supporting institutionalization; presence of universities and research institutions with well-equipped labs.	Strong intersectoral collaboration during emergencies; existing access to EU funding and technical networks; mature institutional and regulatory systems	Strong federal-level political commitment to One Health; establishment of collaborative frameworks among stakeholders; skilled workforce; emerging surveillance capacity; community engagement in health interventions
Weaknesses	Limited domestic funding; unclear institutional roles causing overlaps and inefficiencies; low engagement of animal and environmental sectors; lack of integrated data-sharing systems; administrative & logistical barriers hindering policy translation, especially during government transitions	No formal One Health strategy; weak routine data sharing & collaboration; lack of a centralized One Health platform resulting in fragmented responses and inconsistent integration of environmental data	No dedicated One Health legislation; poor infrastructure; funding constraints and absence of dedicated budget lines; limited private sector engagement; weak data sharing; poor retention of workforce at subnational levels; limited or uneven research capacity and weak uptake of research outputs
Opportunities	Increasing international partnerships (WHO, FAO, Africa CDC); training and workforce development; leveraging technology & AI for disease surveillance and early warning systems	Increasing global and EU funding for One Health research and implementation; regional technical networks; potential to develop a formal One Health curriculum to strengthen future workforce capacity.	Rising donor interest; eco-health and sustainability programs; collaboration with NGOs, academia and private sector to bolster One Health programs; strong potential to leverage political advocacy structures (e.g., NGF, ALGON)
Threats	Political transitions which may disrupt One Health initiatives and policy continuity; climate change; rural service delivery challenges; emerging disease threats which could strain limited resources; increased cross-border population movement and associated health-security risks	Rising AMR; climate-related health risks; continued reliance on emergency-mode coordination	Insecurity in rural areas; weak regulation of animal movement; persistent brain drain; disruption due to political instability; climate change

### 5.2 Prioritization of Strategic Areas for One Health Action

Country-specific SWOT profiles informed facilitated stakeholder deliberations to identify strategic priorities for strengthening national One Health systems.

Although the resulting priorities reflected each country's context, common themes emerged including strengthening governance and institutionalization, integrated surveillance, AMR control, climate resilience, workforce development, sustainable financing and multirsectoral coordination. Croatia additionally prioritized climate adaptation, sustainable agriculture, and One Health education. Ghana emphasized institutionalizing the national One Health Strategy and Action Plan and strengthening pandemic preparedness. Nigeria placed greater emphasis on One Health research, policy implementation, environmental health, risk communication, and community engagement. Stakeholders also identified country-specific knowledge translation topics to support evidence-informed policymaking through the development of Evidence Briefs for Policy (Table 6).

**Table 6:** Country-level strategic priorities and knowledge translation topics for One Health implementation.

Country	Strategic Priority Areas for One Health Action	Potential One Health Topic for Knowledge Translation (e.g. Evidence Briefs for Policy)
Croatia	Strengthening intersectoral data sharing and collaboration	Development of an integrated One Health surveillance system for zoonotic diseases, AMR and environmental health
	Enhancing public and professional awareness of AMR	Knowledge translation on responsible antibiotic use in human and animal health to address AMR
	Strengthening climate change adaptation and vector control	Effective vector management and climate adaptation strategies to reduce vector-borne diseases risks
	Promoting sustainable agricultural practices	Implementation of biosecurity and sustainable pest control practices in agriculture to support One Health goals
	Establishing a national One Health educational curriculum	Curriculum development that includes integrated One Health topics for public health, veterinary and environmental science students
	Developing a formal national One Health coordinating body	Establishing a permanent national coordinating platform to ensure proactive policy implementation and routine intersectoral collaboration

Ghana	Institutionalizing the One Health strategy and Action Plan	Effective implementation of the One Health strategy and legal framework to strengthen collaboration, communication, and surveillance across sectors for early detection and response to health threats
	Strengthening pandemic preparedness and prevention	Enhancing surveillance, diagnostic capacity and preparedness for priority epidemic-prone diseases like Ebola, Dengue Monkey pox, Lassa fever and Marburg
	Enhancing One Health multisectoral collaboration and accountability	Strengthening institutional roles, accountability mechanisms, communication, and routine collaboration across the human, animal and environmental health sectors
	Strengthening zoonotic disease surveillance and response	Developing comprehensive surveillance plans and biosecurity measures for diseases transmissible between animals and human (e.g. anthrax, rabies, zoonotic tuberculosis and highly pathogenic avian influenza)
	Strengthening the national response to AMR	Policy options for strengthening AMR surveillance and monitoring resistance patterns in humans, animals and the environment Improving infection prevention and control practices in healthcare settings and animal production systems
	Integrating One Health approaches into research and educational programs	Investing in research, training and education for professionals in various sectors and universities to promote One Health expertise and generate evidence for policy

Nigeria	Strengthening One Health research	Strengthening national mechanisms for identifying, conducting, funding, synthesizing, and applying priority One Health research
	Strengthening One Health policy development and implementation	Policy options for addressing legal, institutional, and implementation gaps in Nigeria's One Health framework
	Developing a coordinated surveillance framework for zoonotic diseases	Policy options for establishing coordinated multisectoral surveillance of priority zoonotic diseases in Nigeria to enhance public health outcomes
	Developing an integrated AMR surveillance system for priority pathogens	Strengthening integrated AMR surveillance for priority pathogens across the human, animal, and environmental sectors
	Sustaining financial support for One Health and AMR.	Developing an advocacy plan for establishing dedicated domestic financing for One Health and AMR initiatives in Nigeria
	Conducting a One Health prevalence survey for priority zoonotic diseases	Using One Health prevalence survey to strengthen the prevention and control of priority zoonotic diseases in Nigeria
	Strengthening environmental health and sustainability	Policy options for improving conservation and management of natural resources within a One Health framework. Developing and implementing effective environmental health policies and regulatory mechanisms
	Enhancing One Health communication and community engagement	Developing and implementing effective One Health communication strategies. Policy options for improving public communication, community participation and engagement in One Health initiatives among human, animal and environmental health professionals
	Strengthening One health workforce capacity and development	Developing and implementing effective One Health workforce development policies and strategies. Developing and implementing integrated One Health education and training programs for relevant professional groups
	Raising public awareness of the One Health approach	Developing and implementing strategies and guidelines for public awareness of the One Health approach

## 6 Discussion

Despite growing global consensus around One Health, translating the approach into routine implementation remains challenging. This study applied the KOHSRA tool in three countries to assess national readiness for One Health implementation and to identify context-specific priorities for strengthening One Health systems.

Across the three countries, many of the foundational One Health system capacities, including governance arrangements, surveillance and emergency response systems, coordination mechanisms, and sector-specific institutional structures were generally present. However, these capacities remained fragmented, unevenly institutionalized, and were often supported through external initiatives or activated primarily during emergencies rather than embedded within routine national systems. Below, we propose four broad considerations for strengthening One Health systems and advancing evidence-informed One Health implementation.

First, strengthening One Health systems requires governance and coordination arrangements that extend beyond sector-specific policies and existing institutional structures. Croatia had well-established legal and regulatory frameworks for food safety and AMR, but lacked an overarching national One Health strategy and permanent coordination platform. Ghana had developed a draft national One Health strategy and established a multisectoral technical working group, while Nigeria had established a national coordinating unit following implementation of its One Health strategic plan. Nevertheless, One Health implementation was perceived as fragmented, incompletely institutionalized, and constrained by weak legal mandates, limited subnational integration, and the absence of comprehensive national One Health policy and governance frameworks. These findings are consistent with evidence showing that effective One Health implementation requires integrated policy frameworks, formally embedded and adequately resourced multisectoral coordination mechanisms, clear accountability, and effective cross-sectoral leadership [7], [23], [24], [25], [26].

Second, sustainable financing is essential for the long-term implementation and institutionalization of One Health. Across the three countries, One Health activities continued to rely heavily on external funding, including development-partner support in Ghana and Nigeria and EU funding mechanisms in Croatia. While these investments have strengthened national capacities, dependence on external resources limits long-term planning, national ownership, and the continuity of multisectoral collaboration. These findings reinforce evidence that sustained domestic financing and the integration of One Health into routine government planning and budgeting are essential for long-term implementation [7], [27].

Third, environmental health remains insufficiently integrated into national One Health systems. Across all three countries, climate-sensitive diseases, ecosystem degradation, biodiversity loss, and pollution were recognized as important risks, yet environmental considerations were less consistently integrated into governance arrangements, surveillance systems, policy implementation, and stakeholder representation than the human and animal health sectors. This reflects the historical emphasis of One Health on zoonotic diseases and AMR and highlights the need to strengthen environmental governance and representation as the One Health agenda expands to address broader ecological and climate-related challenges [28], [29], [34], [35], [36].

Fourth, strengthening evidence systems (including integrated surveillance, research, knowledge translation, and monitoring and evaluation) together with workforce capacity is critical for evidence-informed One Health policy and implementation. While surveillance and research capacities were present across the three countries, they remained largely sector-specific, with limited interoperability, routine data sharing, and integration of environmental information. In addition, mechanisms for coordinating research agendas and supporting knowledge translation, centralized evidence repositories, integrated monitoring and evaluation, and interdisciplinary One Health education were identified as only partially institutionalized during the assessment. These limitations constrain institutional learning, accountability, and the routine generation, sharing, integration, and use of evidence and data across the human, animal, and environmental sectors. Strengthening integrated evidence systems, interdisciplinary workforce development, and institutionalized mechanisms for knowledge translation and evidence use will be critical for embedding evidence-informed decision-making into routine One Health policy and practice [30], [31], [32], [33].

Beyond these cross-cutting considerations, the priority-setting exercise demonstrated how shared readiness challenges can be translated into context-specific policy priorities. While common priorities centered on strengthening governance and institutionalization, integrated surveillance, AMR control, climate resilience, workforce development, sustainable financing, and intersectoral coordination, each country also identified priorities reflecting its own institutional context and policy needs. These priorities provided country-specific directions for strengthening One Health systems and directly informed the development of Evidence Briefs for Policy within the OH-EVI project, illustrating how structured readiness assessments can translate system gaps into context-specific policy priorities and implementation options.

The pilot application of KOHSRA in three countries provides initial evidence of its operational feasibility and potential utility as a structured framework for assessing national One Health systems readiness and translating assessment findings into strategic priorities for policy and implementation. As a participatory assessment based on structured, facilitated stakeholder consensus, the framework supports contextual assessments and priority setting rather than objective evaluations or rankings of national performance. Nevertheless, it provide valuable insights into system strengths, implementation gaps, and priority areas for strengthening One Health systems. Additional applications across diverse settings, together with formal validation studies, are warranted to further evaluate the framework's reliability, validity, reproducibility, and broader applicability, thereby strengthening its contribution to evidence-informed One Health implementation.

## 7 Limitations

Several potential limitations should be considered when interpreting the findings.

First, implementation of the KOHSRA tool relied on a structured participatory process requiring the engagement of multisectoral stakeholders. Consequently, the findings primarily reflect facilitated stakeholder consensus and should therefore be interpreted as contextual assessments of perceived system readiness rather than objective evaluations of national performance. As with any self-assessment, the process remains susceptible to institutional and social desirability bias. To improve consistency, participants were encouraged to support their assessments with documentary evidence, and discussions were guided using a standardized facilitation approach.

Additionally, to facilitate cross-country comparison and visualization, findings from Components 1 and 4 were recoded into three standardized reporting categories (“Available,” “To some extent,” and “Absent”). While these categories enabled broad cross-country comparison, they may have obscured differences in the quality, maturity, or scope of similarly classified arrangements and should therefore not be interpreted as rankings of national performance. To preserve contextual nuance, the standardized categories were interpreted alongside participants' narrative justifications and supporting documentary evidence.

Finally, although KOHSRA has undergone expert review and pilot testing, it has not been formally evaluated for content validity, reliability, or construct validity. The tool is intended primarily as a systems assessment and facilitation framework rather than a conventional psychometric measurement instrument. Future research is warranted to further evaluate the framework's reliability, validity, reproducibility, and applicability across a broader range of countries and implementation settings. In addition, repeated applications will be important to assess changes in system readiness over time and further evaluate the framework's utility for supporting evidence-informed decision-making.

## 8 Conclusion

The K2P One Health Readiness Assessment (KOHSRA) Tool provides a pragmatic, systems-oriented approach for assessing national readiness and identifying strategic priorities for strengthening One Health. Its application in Croatia, Ghana and Nigeria highlighted the importance of formal governance structures, routine multisectoral coordination, sustainable domestic financing, interoperable information systems and institutionalized evidence-informed policymaking for effective One Health implementation. These findings offer governments and development partners a practical basis for aligning technical assistance and investments with country-defined priorities.

Beyond its application in the three countries, KOHSRA offers a transferable framework that can support evidence-informed policy development and multisectoral health system strengthening across diverse settings. Future applications in additional countries, together with formal validation studies, will help refine the framework and further evaluate its reliability, validity, reproducibility, and applicability across different governance and health system contexts.

## CRediT authorship contribution statement

**Fadi El-Jardali:** Writing – review & editing, Writing – original draft, Supervision, Resources, Methodology, Funding acquisition, Conceptualization. **Racha Fadlallah:** Writing – review & editing, Writing – original draft, Visualization, Supervision, Project administration, Methodology, Formal analysis, Data curation, Conceptualization. **Francis Sena Nuvey:** Writing – review & editing, Resources, Project administration, Funding acquisition, Data curation, Conceptualization. **Nadine Darwiche:** Writing – review & editing, Writing – original draft, Visualization, Methodology, Investigation, Formal analysis, Data curation. **Barbara Bekavac:** Writing – review & editing, Validation, Methodology, Investigation, Formal analysis, Data curation. **Pavle Jeličić:** Writing – review & editing, Validation, Investigation, Formal analysis, Data curation. **Luka Delak:** Writing – review & editing, Validation, Investigation, Formal analysis. **Brigita Hengel:** Writing – review & editing, Validation, Methodology, Formal analysis, Data curation. **John Humphrey Amuasi:** Writing – review & editing, Validation, Supervision, Investigation, Formal analysis, Data curation. **Leslie Mawuli Aglanu:** Writing – review & editing, Validation, Formal analysis, Data curation. **Daniel Opoku:** Writing – review & editing, Validation, Formal analysis, Data curation. **Jesse Uneke:** Writing – review & editing, Validation, Supervision, Investigation, Formal analysis, Data curation. **Onyedikachi Chukwu:** Writing – review & editing, Validation, Formal analysis, Data curation. **Amos Enyi:** Writing – review & editing, Validation, Data curation. **Davi Romão:** Writing – review & editing, Resources, Project administration, Conceptualization. **Tanja Kuchenmüller:** Writing – review & editing, Resources, Project administration, Funding acquisition, Conceptualization. **Anna Fahrion:** Writing – review & editing, Resources, Project administration, Methodology, Funding acquisition, Conceptualization.

## Funding

The Knowledge to Policy (K2P) Center at the American University of Beirut (AUB) supported the development of the KOHSRA tool. The piloting of the work was funded by the German Federal Ministry of Health (BMG) through the Global Health Protection Programme (GHPP) and implemented by the Friedrich-Loeffler-Institut (FLI) in collaboration with the World Health Organization (WHO) under the project Evidence-informed Policy Approaches for One Health (OH-EVI) [ZMI5-2523GHP012]. The funders had no role in the study.

## Declaration of competing interest

The authors declare the following financial interests/personal relationships which may be considered as potential competing interests:

Francis Sena Nuvey reports financial support was provided by German Federal Ministry of Health. If there are other authors, they declare that they have no known competing financial interests or personal relationships that could have appeared to influence the work reported in this paper.

## Data Availability

Data will be made available on request.

## Appendix A Supplementary data

Supplementary File 1. Additional Methodological Details for KOHSRA Tool Development
