# Supplementary material for: Strengthening one health systems: A readiness assessment across Croatia, Ghana and Nigeria
Source: One Health. 2026 Jul 28;23:101533. doi: 10.1016/j.onehlt.2026.101533 (PMC13470580; doi:10.1016/j.onehlt.2026.101533)
Supplement: Supplementary file 1 [file mmc1.docx]

Supplementary File 1. Additional Methodological Details for KOHSRA Tool Development

#### **Literature-Derived Parameters**

The following core assessment parameters were identified from the literature review:

- Governance and policy (existence of formal mandates, national strategies, and enabling policies that institutionalize One Health coordination)
- Multisectoral coordination and collaboration (mechanisms for inter-agency communication, joint planning, and shared accountability across human, animal, and environmental sectors)
- Surveillance, information sharing, and data systems (integration of surveillance networks and data exchange platforms for early detection and response)
- Laboratory and diagnostic capacity (cross-sector laboratory collaboration, standardized protocols, and quality assurance systems)
- Workforce development and training (interdisciplinary training, workforce capacity, and professional exchange across sectors0
- Risk assessment, preparedness, and response (joint risk assessments, emergency preparedness planning, and simulation exercises)
- Environmental and ecosystem health integration (mechanisms to address environmental drivers of health threats, such as land use, pollution, and climate change)
- Research, innovation, and evidence generation (national research priorities, knowledge exchange, and translation of evidence into policy)
- Communication and community engagement (risk communication, stakeholder engagement and community-based One Health initiatives)
- Financing and sustainability (dedicated resources and long-term investment strategies to sustain One Health initiatives)

**Key Distinctions Between KOHSRA and Existing One Health Operational Tools**

| Dimension | Existing Tools | KOHSRA Tool |
| --- | --- | --- |
| Coverage across One Health system components | Selected tools often focus on specific functions or domains (e.g., OHZDP for prioritization, OH-SMART for multisectoral coordination, SIS-OT for surveillance). | Provides a system-wide perspective covering policy, governance, institutional capacity, surveillance, data, research, community engagement, monitoring, environment, and policy analysis. |
| Inclusion of all three sectors (human, animal, environment) | Some tools give greater emphasis to human and animal health, with comparatively less attention to environmental dimensions (e.g., WHO-WOAH NBW). | Explicitly integrates environmental health across indicators and data analysis. |
| Strategic Prioritization | Several tools focus mainly on assessment, mapping, facilitation, or prioritization without a structured process for translating findings into strategic action. | Adds a dedicated prioritization phase using SWOT-informed analysis to support identification and prioritization of strategic actions and development of country-specific Evidence Briefs for Policy. |
| Participatory approach | Many tools use expert-led or facilitated participatory processes. | Uses a structured national self-assessment process to promote stakeholder ownership and consensus. |
| Evidence-informed policy lens | Few tools explicitly examine evidence-informed policymaking mechanisms or their links to One Health governance. | Includes Evidence-Informed Policymaking and policy coherence components to connect technical capacity with decision-making. |
| Cross-Sector Policy Coherence | Many tools focus on technical capacities without systematically assessing alignment across sectoral policies. | Includes a dedicated component examining coherence across One Health, public health, environmental, and agricultural policies to support integrated implementation. |
| Adaptability beyond outbreak preparedness | Several tools were developed primarily for zoonotic disease, emergency preparedness, or specific technical functions. | Designed to support broader One Health system readiness assessment, including governance, policy coherence, institutional strengthening, and long-term multisectoral coordination beyond emergency response. |
